# Cooperative Design of Ranging and Communication for In-Band Full-Duplex Inter-Satellite Links

**DOI:** 10.3390/s26103013

**Published:** 2026-05-10

**Authors:** Hao Feng, Zhuo Yang, Hong Ma, Yiwen Jiao, Tao Wu, Yang Cai, Hongbin Ma, Zhiyong Shan

**Affiliations:** 1 Space Engineering University, Beijing 101416, China; fenghao123@hgd.edu.cn (H.F.); mahong_0108@163.com (H.M.); jiaoyiwen1985@163.com (Y.J.); wt_ttco2023@163.com (T.W.); caiyang_1991@163.com (Y.C.); hongbin_ma@163.com (H.M.); wazgjss@163.com (Z.S.); 2 Key Laboratory of Intelligent Space TTC&O, Ministry of Education, Beijing 101416, China

**Keywords:** BDS-3, inter-satellite link, in-band full-duplex, communication efficiency, signal acquisition

## Abstract

**Highlights:**

**What are the main findings?**

- With the assistance of broadcast ephemeris, the signal acquisition time is compressed to the millisecond level, and signal tracking is completed within a 250 ms preamble while maintaining the ranging accuracy unchanged.
- A cooperative design of ranging and communication for BDS inter-satellite links based on an in-band full-duplex system is proposed, achieving a 5-fold improvement in communication efficiency through time-slot reconfiguration.

**What are the implications of the main findings?**

- This work provides a new time-slot resource allocation framework for inter-satellite links, enabling cooperative optimization of ranging and communication without changing the existing 3 s time-slot structure
- The proposed method can be deployed as an enhanced mode in next-generation navigation constellations, supporting autonomous constellation operation and high-capacity inter-satellite data transmission.

**Abstract:**

To address the limited communication capacity of the traditional time-division half-duplex (TDHD) systems in BDS-3 inter-satellite links (ISLs), this paper proposes a cooperative design of ranging and communication based on an in-band full-duplex (IBFD) architecture. By utilizing BDS broadcast ephemeris to assist signal acquisition and selecting the serial acquisition strategy with the lowest computational complexity, a 100% acquisition success rate can be achieved within milliseconds. This completely releases the 250 ms preamble originally used for acquisition in the traditional time slot. Adopting the IBFD system, the ISL time-slot structure is optimally redesigned: the preamble is used for signal acquisition and tracking to accomplish inter-satellite ranging, while the original measurement period is used for QPSK dual-channel parallel data transmission. This design extends the effective communication duration from 1 s to 2.5 s, expands the communication from single-channel to dual-channel, and theoretically achieves a 5-fold improvement in communication efficiency. Simulation analysis shows that, while the communication efficiency is significantly improved, the ranging accuracy remains essentially unchanged compared with the traditional TDHD system. Without altering the existing 3 s time-slot duration, this method achieves cooperative optimization of ranging and communication, providing a feasible technical solution for enhancing the communication capacity of BDS-3 ISLs.

## 1. Introduction

As a key technology for autonomous operation and cooperative networking of satellite constellations, inter-satellite links (ISLs) have attracted extensive attention from major spacefaring nations in recent years [1,2,3]. Through ISLs, satellites can directly perform high-precision ranging and data exchange with each other, enabling autonomous inter-satellite navigation, thereby significantly reducing dependence on ground tracking stations and improving the autonomous survivability and global service continuity of the constellation [4,5]. In navigation systems, ISLs have been successfully applied to tasks such as autonomous orbit determination, inter-satellite time synchronization, satellite-ground integrated precise orbit determination, and inter-satellite communication transmission, becoming an important infrastructure supporting high-performance services of the system [6,7,8].

Following the three-step development strategy, China’s BDS-3 has evolved from a regional system to a global system [9]. The core challenge lies in the difficulty of deploying ground stations worldwide, making real-time monitoring and orbit determination of overseas satellites a major technical bottleneck [10,11]. The breakthrough in ISL technology is the key to solving this problem. In 2015, the first BDS-3 satellite equipped with an ISL payload was successfully launched [12]. By 31 July 2020, the BDS-3 global system was officially commissioned, and the ISL has since achieved stable in-orbit operation [13]. The BDS-3 ISL adopts Ka-band phased array technology, which enables each satellite to flexibly control its beam pointing via the phased array antenna, achieving fast acquisition and stable tracking of ISLs and supporting high-dynamic networking communication among constellation satellites [14,15].

Currently, BDS-3 ISLs have become a research hotspot among scholars worldwide, with research efforts primarily covering key technologies such as high-precision inter-satellite ranging, autonomous orbit determination, time synchronization, and routing networking [16]. In terms of inter-satellite ranging, Zhang et al. analyzed the ranging performance of BDS-3 ISLs based on 30 days of ISL observation data. The results showed that the RMS error of all links was approximately 2.5 cm, and different satellite manufacturers had a significant impact on the measurement error [17]. Guo et al. systematically investigated various errors and their correction methods in the two-way measurement process of ISLs, and the ranging results indicated that the ISL measurement accuracy was approximately 7 cm [18]. In terms of autonomous orbit determination, Li et al. proposed an autonomous orbit determination method for BDS-3 satellites that integrates satellite-ground, inter-satellite, and space-pointing observations. Experimental results showed that a three-dimensional orbit position accuracy of 0.45 m could be achieved after 90 days of autonomous operation in an inertial frame [19]. Xia et al. performed distributed autonomous orbit determination using a sequential Kalman filter algorithm based on 180 days of ISL observation data. Under precise Earth orientation parameter (EOP) conditions, the three-dimensional position error and user range error (URE) of MEO satellites reached 2.1 m and 0.43 m, respectively [20].

With respect to time synchronization and routing networking, Yan et al. proposed an autonomous clock synchronization and time keeping method for BDS-3. Experimental analysis based on 30 days of observation data from 29 BDS satellites showed that the method could reduce time drift to 2.6 ns within 60 days, achieving a daily frequency stability of 8.4 × 10^−16^ [21]. Guo et al. proposed a two-way time synchronization method based on BDS-3 Ka-band satellite-ground link observations. By estimating relative clock bias and correcting error terms such as hardware delays, experimental results demonstrated that the satellite-ground time synchronization accuracy of the method was better than 0.3 ns [22]. Wang et al. proposed a cooperative allocation scheme for hybrid laser and microwave inter-satellite links, constructed a multi-objective optimization model, and designed an allocation algorithm based on NSGA-II and genetic algorithms. The effectiveness of the scheme in improving constellation autonomous orbit determination and data transmission performance was verified [23]. Yan et al. proposed an optimization method for GNSS ISL allocation based on rolling weight matching (RWM). Experimental results from BDS-3 constellation simulations over 10,080 superframes showed that the RWM method achieved an average of 17.28 ISLs per satellite [24].

Current research is primarily conducted based on actual observation data under the existing BDS-3 ISL architecture, while innovative research targeting the ISL architecture itself remains relatively scarce. BDS-3 ISLs adopt a time-division half-duplex (TDHD) architecture, in which transmission and reception are time-separated within each 3 s time slot, each occupying 1.5 s [25]. This architecture suffers from several limitations, including high complexity in ISL establishment, limited duration for both ranging and communication, and significant time-scale conversion errors.

In recent years, in-band full-duplex (IBFD) technology has achieved breakthrough progress and has attracted widespread attention from both academia and industry, providing an effective technical pathway for improving ISL performance. IBFD technology enables wireless devices to transmit and receive signals simultaneously on the same frequency. Compared with time-division duplex (TDD) systems, it can approximately double the information throughput within the same time interval. Compared with frequency-division duplex (FDD) systems, it effectively saves bandwidth resources and improves spectral efficiency. IBFD technology has been extensively studied in areas such as massive MIMO systems, low-power wide-area networks (LPWANs), and relay communication networks, where substantial technical experience has been accumulated. Introducing IBFD technology into ISL scenarios can effectively simplify the link establishment process, eliminate the segmentation of measurement duration inherent in TDHD systems, and improve both ranging and communication performance of ISLs [26,27,28].

Under the IBFD architecture, the target signal is subject to self-interference from the locally transmitted high-power signal, which prevents the communication system from correctly extracting the desired signal. Therefore, effectively suppressing self-interference is a key technical challenge that must be addressed to realize IBFD systems. From the perspective of where self-interference suppression is performed, existing research has mainly focused on three domains: spatial domain, radio frequency (RF) domain, and digital domain. In the spatial domain, various methods such as spatial isolation, phase adjustment, coupling network design, and artificial material applications are typically adopted. In the RF and digital domains, the primary strategy is to reconstruct the self-interference channel and perform cancellation [29,30].

Based on the analysis of topological characteristics of the BDS-3 constellation under satellite visibility and antenna pointing constraints, four types of ISLs can be identified: MEO same-orbit links, MEO same-plane inter-orbit links, MEO-to-GEO/IGSO links, and links between high-orbit satellites. The maximum inter-satellite distance reaches 68,600 km, the minimum distance is 27,900 km, and the maximum relative velocity between satellites reaches ±3.8 km/s, resulting in an approximately 8 dB variation in the received carrier-to-noise ratio (C/N_0_) [31]. The BDS-3 ISLs adopt QPSK digital modulation schemes. Link budget calculations under the maximum inter-satellite distance show that the C/N_0_ of the ISL signal is approximately 57.65 dB, which can support at least 20 kbps data transmission with a bit error rate (BER) of 10^−7^. Therefore, this link budget sensitivity must be fully considered in the design of IBFD-based ISLs.

In our previous work, the feasibility of applying IBFD technology to ISL scenarios has been demonstrated. In practical BDS-3 scenarios, the required self-interference suppression for full-duplex operation is approximately 175 dB, and current joint self-interference suppression techniques in the spatial, radio frequency, and digital domains can achieve more than 180 dB of suppression [32]. In the spatial domain, spatial isolation and artificial electromagnetic bandgap structures can be employed to achieve effective suppression of most self-interference signals. In the RF domain, a digitally assisted RF signal reconstruction method can be used to achieve signal cancellation. In the digital domain, the adaptive filtering algorithm can be utilized to estimate and reconstruct the self-interference channel, thereby enabling self-interference signal cancellation. Building on this foundation, this paper optimizes the design of the BDS-3 ISL measurement and communication architecture based on the IBFD system, improving communication efficiency by approximately fivefold while maintaining ranging accuracy essentially unchanged.

The remainder of this paper is organized as follows. Section 2 presents the fast inter-satellite acquisition method assisted by BDS broadcast ephemeris. Section 3 presents the design of the measurement and communication architecture based on the IBFD system. Section 4 conducts simulation validation, analyzing ephemeris calculation accuracy, fast acquisition performance, and inter-satellite measurement accuracy. Section 5 concludes the paper and provides an outlook for future work.

## 2. Fast Inter-Satellite Acquisition Method Assisted by Broadcast Ephemeris

Assuming the ISL carrier frequency is 23 GHz, the spreading code rate is 10.23 MHz, and the code length is 1023 chips [33]. The signal propagation delay range can be calculated as [93 ms, 228.67 ms], and the Doppler frequency shift range as [−291.33 kHz, 291.33 kHz]. Typically, the code phase acquisition search step size $\tau_{\mathrm{step}}$ is 0.5 chips, and the Doppler frequency search step size $f_{\mathrm{step}}$ is $2/3T_{\mathrm{coh}}$, where $T_{\mathrm{coh}}$ is the coherent integration duration. When one code period is selected as the coherent integration duration, the number of code phase search bins is 2046, the number of Doppler frequency search bins is approximately 89, and the total number of search bins reaches as high as 182,094, making it difficult to achieve fast signal acquisition within a short time.

### 2.1. Satellite State Calculation with Broadcast Ephemeris

BDS-3 satellites store their own broadcast ephemeris. After ISL networking and establishment, satellites can use the data transmission function of ISLs to broadcast their ephemeris to the entire constellation. Therefore, during the signal acquisition phase, the broadcast ephemeris of both the transmitting and receiving satellites can be used to calculate satellite positions and velocities, thereby reducing the uncertainty of time delay and Doppler frequency, significantly decreasing the search range and computational complexity of signal acquisition, and meeting the requirements for fast acquisition of inter-satellite link signals.

Based on the BDS broadcast ephemeris, the position vector of BDS-3 MEO and IGSO satellites in the Earth-Centered, Earth-Fixed (ECEF) coordinate system can be calculated as:

(1) $${\dot{r}}_{\mathrm{ECEF}}=R_{\mathrm{orb}}^{\mathrm{ECEF}}r_{\mathrm{orb}}$$

where $r_{\mathrm{orb}}$ represents the position vector of the BDS-3 satellite in the orbital coordinate system, and $R_{\mathrm{orb}}^{\mathrm{ECEF}}$ represents the rotation matrix from the orbital coordinate system to the ECEF coordinate system. It can be further expanded as:

(2) $$R_{\mathrm{orb}}^{\mathrm{ECEF}}=R_{z}(-\Omega_{k})\cdot R_{x}(-i_{k})$$

(3) $$r_{\mathrm{orb}}=\left[\begin{array}{c} r_{k}\cos u_{k}\\ r_{k}\sin u_{k}\\ 0 \end{array}\right]$$

where $r_{k}$ represents the BDS-3 satellite’s radial distance at observation epoch k, $u_{k}$ represents the satellite’s argument of latitude, $\Omega_{k}$ represents the right ascension of the ascending node, $i_{k}$ represents the orbital inclination, and $R_{z}$ and $R_{x}$ are the rotation matrices around the Z-axis and X-axis, respectively. The velocity vector of the BDS-3 MEO and IGSO satellites can be obtained by differentiating Equation (1):

(4) $${\dot{r}}_{\mathrm{ECEF}}={\dot{R}}_{\mathrm{orb}}^{\mathrm{ECEF}}r_{\mathrm{orb}}+R_{\mathrm{orb}}^{\mathrm{ECEF}}{\dot{r}}_{\mathrm{orb}}$$

For BDS-3 GEO satellites, due to their geostationary nature, an additional rotation needs to be applied to the initially calculated coordinates to obtain their true positions. The calculation formula is as follows:

(5) $$r_{\mathrm{ECEF}}=R_{z}({\dot{\Omega }}_{k}t_{k})\cdot R_{x}(-5{}^{\circ})\cdot (R_{\mathrm{orb}}^{\mathrm{ECEF}}r_{\mathrm{orb}})$$

where $t_{k}$ represents the calculation epoch, and ${\dot{\Omega }}_{k}$ represents the rate of change of the right ascension of the ascending node. Differentiating Equation (5) yields the velocity vector of the GEO satellite:

(6) $${\dot{r}}_{\mathrm{ECEF}}={\dot{R}}_{z}({\dot{\Omega }}_{k}t_{k})\cdot R_{x}(-5{}^{\circ})\cdot (R_{\mathrm{orb}}^{\mathrm{ECEF}}r_{\mathrm{orb}})+R_{z}({\dot{\Omega }}_{k}t_{k})\cdot R_{x}(-5{}^{\circ})\cdot ({\dot{R}}_{\mathrm{orb}}^{\mathrm{ECEF}}r_{\mathrm{orb}}+R_{\mathrm{orb}}^{\mathrm{ECEF}}{\dot{r}}_{\mathrm{orb}})$$

### 2.2. Acquisition Strategy Design with Ephemeris Assistance

Signal acquisition methods can be broadly categorized into three types: serial acquisition, code-parallel acquisition, and frequency-parallel acquisition. With broadcast ephemeris assistance, the satellite velocity calculation accuracy is very high, typically within 1 mm/s, resulting in a Doppler frequency uncertainty of less than 1 Hz. Consequently, Doppler frequency search is unnecessary, and only serial acquisition and code-parallel acquisition need to be considered. The computational complexities of these two methods are analyzed below.

#### 2.2.1. Serial Acquisition

**Figure 1:**
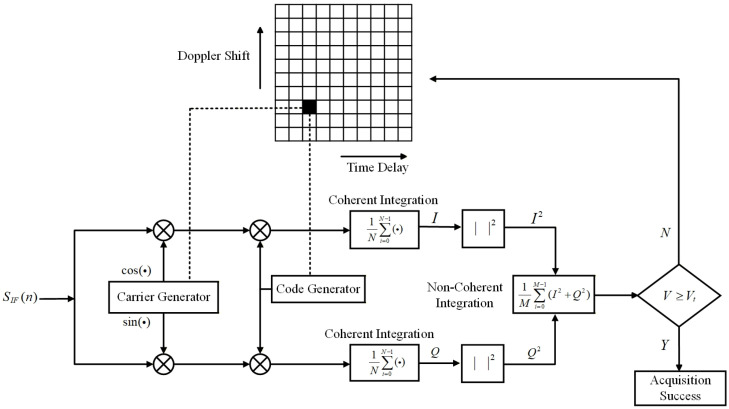
Schematic diagram of serial acquisition.

Serial acquisition is the most fundamental search strategy, also known as signal acquisition based on a time-domain correlator [34]. Figure 1 shows a schematic diagram of the serial acquisition principle. The input signal $S_{\mathrm{IF}}(n)$ is the digital intermediate frequency signal obtained after down-conversion and ADC sampling of the inter-satellite received signal. It is first multiplied by the locally generated carrier and pseudo-code. The multiplication results are then coherently accumulated, with the number of coherent accumulations denoted as N. Let the sampling frequency be $f_{s}$, then the coherent integration duration $T_{\mathrm{coh}}$ can be expressed as $N/f_{s}$. To further improve the signal-to-noise ratio and eliminate the influence of carrier phase error, the output results are typically squared after coherent accumulation, followed by non-coherent accumulation, with the number of non-coherent accumulations denoted as M. The output is used as a decision metric. When the decision metric exceeds a preset threshold, signal acquisition is achieved. Otherwise, the search bin is changed, the local carrier frequency and pseudo-code phase are adjusted, and the acquisition process is repeated.

According to the serial acquisition procedure, a single search requires 2N−1 additions and 4N+2 multiplications. Among these, the 2N−1 additions consist of 2N−2 additions for coherent integration and 1 addition for magnitude accumulation, while the multiplications consist of 4N multiplications for multiplication with the local carrier and pseudo-code, and 2 multiplications for magnitude calculation. When non-coherent accumulation is considered, an additional M−1 additions are required. Let the uncertainty ranges of time delay and Doppler frequency be Δτ and $\Delta f_{d}$, respectively. Then the numbers of search bins for time delay and Doppler frequency, denoted as $N_{\tau }$ and $N_{f_{d}}$, can be expressed as $\left\lceil \Delta \tau /\tau_{\mathrm{step}}\right\rceil$ and $\left\lceil \Delta f_{d}/f_{\mathrm{step}}\right\rceil$, respectively, where $\left\lceil •\right\rceil$ denotes the ceiling function. Therefore, the total computational load of serial acquisition is $[(2N-1)M+(M-1)]N_{\tau }N_{f_{d}}$ additions and $(4N+2)MN_{\tau }N_{f_{d}}$ multiplications, and the number of additions can be simplified to $(2NM-1)N_{\tau }N_{f_{d}}$.

#### 2.2.2. Code-Parallel Acquisition

**Figure 2:**
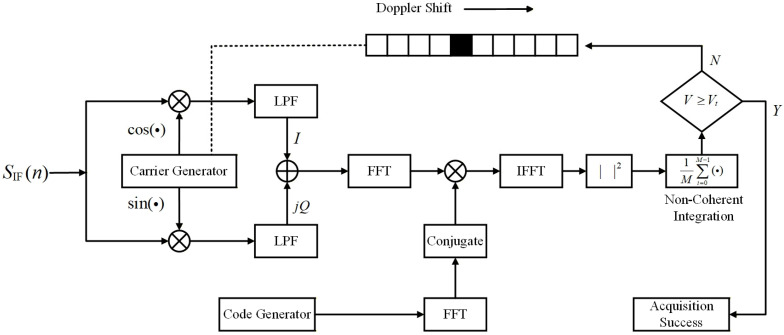
Schematic diagram of code-parallel acquisition.

The code-parallel acquisition algorithm is implemented based on the FFT principle. After carrier stripping of the input signal, the correlation results of the code phase are obtained through two FFT operations and one IFFT operation to achieve parallel processing in the code domain. When N is not an integer power of two, zero-padding is required to extend it to $2^{n}$. Here it is assumed that N can be expressed as $2^{n}$. According to the schematic diagram of the code-parallel acquisition algorithm shown in Figure 2, a single search process requires two FFTs and one IFFT. One FFT or IFFT operation requires $N\log_{2}N$ complex additions and $(N/2)\log_{2}N$ complex multiplications, which correspond to $4N\log_{2}N$ real additions an $2N\log_{2}N$ real multiplications. In addition, carrier stripping involves 2N real multiplications, frequency domain multiplication involves 4N real multiplications and 2N real additions, and magnitude calculation involves 2N real multiplications and N real additions. When non-coherent accumulation is considered, an additional (M−1)N real additions are required. Based on the above analysis, the total computational load of this algorithm is $(6N\log_{2}N+8N)MN_{f_{d}}$ multiplications and $[(12N\log_{2}N+4N)M-N]N_{f_{d}}$ additions.

Based on the BDS broadcast ephemeris, the satellite position and velocity calculation accuracy can be quantitatively evaluated. The derived position and velocity errors determine the uncertainty regions of time delay and Doppler frequency. The computational complexities of serial acquisition and code-parallel acquisition are compared to determine the more efficient strategy. A detailed quantitative analysis of the ephemeris calculation accuracy and the resulting computational complexity will be further presented in the Section 4.

## 3. Measurement and Communication Architecture in IBFD

The introduction of IBFD technology changes the traditional time-slot partitioning of inter-satellite measurement. This section presents the design of the measurement and communication architecture based on the IBFD system. First, the two-way measurement model in IBFD is introduced to describe how simultaneous two-way ranging is achieved. Then, the time-slot structure is redesigned to fully utilize the preamble and improve communication efficiency while maintaining ranging accuracy.

### 3.1. Two-Way Measurement Model

**Figure 3:**
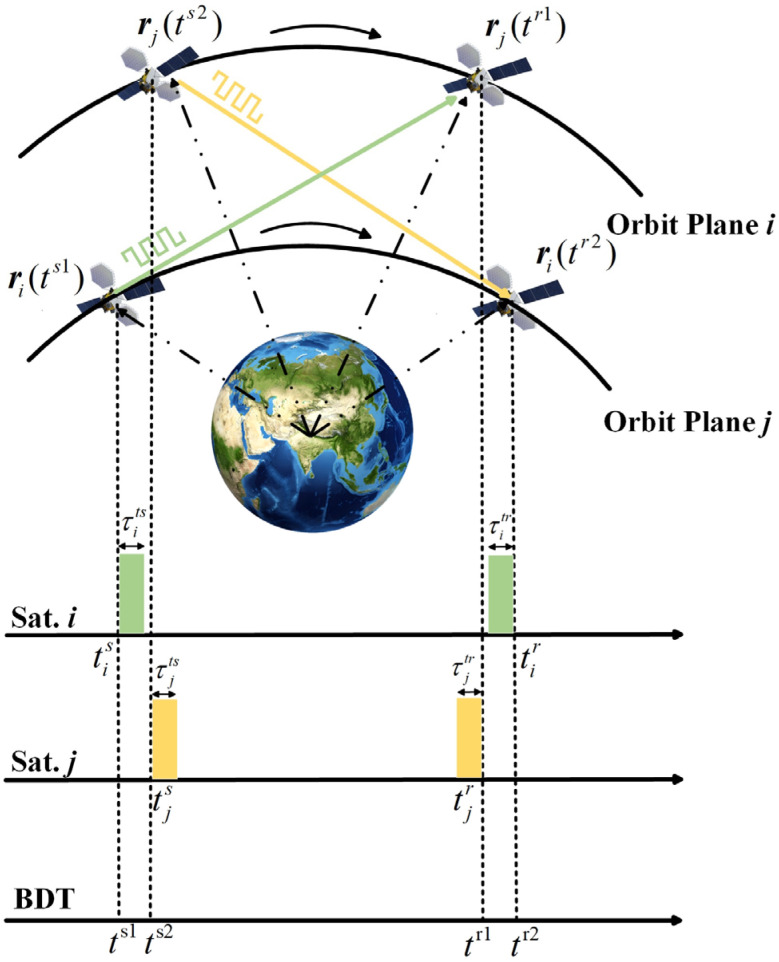
IBFD-based Inter-satellite two-way measurement timing diagram.

Under the IBFD architecture, ISLs can achieve simultaneous transmission and reception, meaning that the two link-establishing satellites send measurement signals to each other at their own local clock time. Figure 3 illustrates the timing diagram of IBFD-based inter-satellite two-way measurement. The two-way measurement process can be expressed as follows:

(7) $$\begin{array}{l} \rho_{ij}=|r_{j}(t^{r1})-r_{i}(t^{s1})|+c[dt_{j}(t^{r1})-dt_{i}(t^{s1})]+c(\tau_{i}^{ts}+\tau_{j}^{tr}+\tau_{ij}^{rel}+\tau_{ij}^{pco})+\varepsilon_{ij}\\ \rho_{ji}=|r_{i}(t^{r2})-r_{j}(t^{s2})|+c[dt_{i}(t^{r2})-dt_{j}(t^{s2})]+c(\tau_{j}^{ts}+\tau_{i}^{tr}+\tau_{ji}^{rel}+\tau_{ji}^{pco})+\varepsilon_{ji} \end{array}$$

where $\rho_{ij}$ and $\rho_{ji}$ are the two-way inter-satellite pseudorange measurements, $dt_{i}(t^{s1})$ and $dt_{i}(t^{r2})$ are the position vectors of satellite i at the transmission and reception epochs, $dt_{j}(t^{s2})$ and $dt_{j}(t^{r1})$ are the position vectors of satellite j at the transmission and reception epochs, $\tau_{i}^{ts}$ and $\tau_{i}^{tr}$ are the transmission and reception channel delays of satellite i, $\tau_{j}^{ts}$ and $\tau_{j}^{tr}$ are the transmission and reception channel delays of satellite j, $\tau_{ij}^{rel}$ and $\tau_{ji}^{rel}$ are the time delay errors caused by relativistic effects in the two-way measurement process, $\tau_{ij}^{pco}$ and $\tau_{ji}^{pco}$ are the phase center offset delays, and $\varepsilon_{ij}$ and $\varepsilon_{ji}$ are the two-way measurement noise.

In the traditional TDHD architecture, two-way inter-satellite measurement requires time-scale conversion to align the bidirectional measurements to the same transmission epoch. This process inevitably introduces time-scale conversion errors, which constitute a significant error source in high-precision inter-satellite ranging. Under the IBFD architecture, the two link-establishing satellites transmit signals simultaneously at their own local clock time, and the clock offset can be determined within 1 ns through inter-satellite time synchronization [18]. Therefore, the need for time-scale conversion is eliminated, and the ranging accuracy is effectively improved.

### 3.2. Time-Slot Structure Design

**Figure 4:**
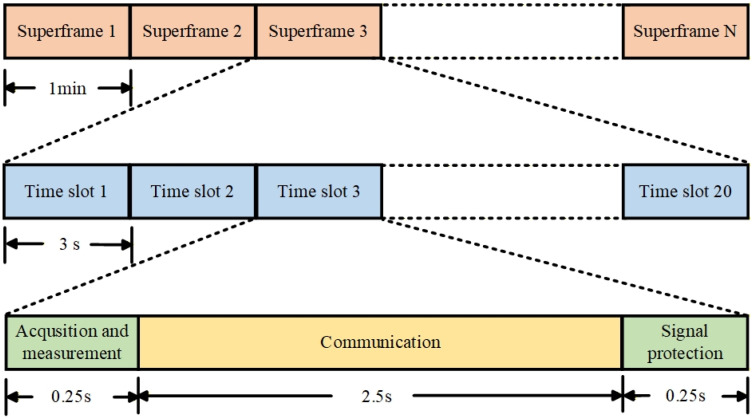
The time-slot architecture based on the IBFD system.

In the TDHD ISL architecture, the 3 s time slot is divided into two halves, with the first 1.5 s for forward transmission and the second 1.5 s for backward transmission. Within each 1.5 s interval, 0.25 s is reserved as a preamble for signal acquisition [35], 0.25 s for a guard interval [36], and only 1 s is left for effective measurement and communication. The BDS-3 ISLs adopt adopts QPSK modulation, where the I branch is used for measurement and the Q branch for communication [37]. As a result, the effective communication duration is only 1 s within a 3 s time slot, which limits communication efficiency and cannot meet the requirements of future high-capacity inter-satellite data transmission. Therefore, this paper optimizes the time-slot architecture based on the IBFD system, as illustrated in Figure 4.

Keeping the time slot duration unchanged at 3 s, the proposed IBFD architecture divides the time slot into three parts: a 0.25 s signal acquisition and tracking period, a 2.5 s communication period, and a 0.25 s guard interval. With the assistance of the BDS broadcast ephemeris, the inter-satellite link acquisition time can be significantly reduced, so most of the 0.25 s period can be used for signal tracking and measurement. During this period, BPSK modulation is innovatively adopted, which provides a 3 dB improvement in carrier-to-noise ratio compared with single-channel QPSK measurement. This higher signal quality compensates for the reduction in tracking duration and ensures that the ranging accuracy remains essentially unchanged. During the communication period, QPSK modulation is adopted, and both the I and Q channels are used for inter-satellite data transmission. Compared with the TDHD architecture, which provides only 1 s of single-channel communication, the proposed architecture achieves approximately a 5-fold improvement in communication efficiency, enabling high-capacity inter-satellite data transmission.

## 4. Simulation and Analysis

To validate the proposed method, three sets of simulation experiments are carried out. First, the calculation accuracy of the BDS broadcast ephemeris is evaluated to provide a basis for determining the uncertainty regions of time delay and Doppler frequency. Based on this, the fast acquisition performance is simulated and analyzed to obtain the acquisition time. Finally, using the acquisition time as a reference, the ranging performance of the proposed IBFD architecture is evaluated and compared with the traditional TDHD architecture to verify that the ranging accuracy remains unchanged while the communication efficiency is significantly improved.

### 4.1. Broadcast Ephemeris Calculation Accuracy Analysis

#### 4.1.1. Data Source and Processing Method

The broadcast ephemeris data of BDS from 12 August to 14 August 2024 (DOY 225–227) are selected in this paper, and the precise ephemeris data released by the Multi-GNSS Data Processing Center of Wuhan University are used as the reference truth value.

Since the broadcast ephemeris provides data at intervals of 2 h, while the precise ephemeris provides orbit data at intervals of 5 min, to reduce the influence of data interpolation on satellite position accuracy, the broadcast ephemeris is used to output satellite coordinates and velocities at 5 min intervals, which are then aligned in time with the precise ephemeris. The satellite coordinates from the precise ephemeris are taken as the true values to evaluate the accuracy of satellite positions calculated from the broadcast ephemeris.

Since the precise ephemeris does not provide satellite velocity information, three days of consecutive precise ephemeris data are concatenated, and a 12th-order sliding Lagrange interpolation method is applied to compute the satellite velocities at the corresponding epochs. The interpolated velocities are used as the reference truth values to evaluate the accuracy of satellite velocities calculated from the broadcast ephemeris.

#### 4.1.2. Calculation Error Evaluation

**Figure 5:**
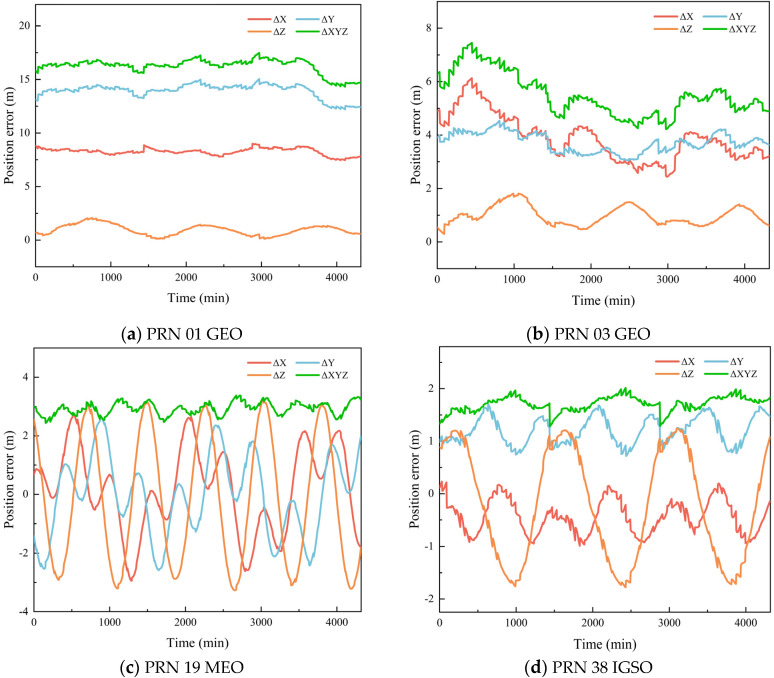
Position calculation error of BDS broadcast ephemeris.

**Figure 6:**
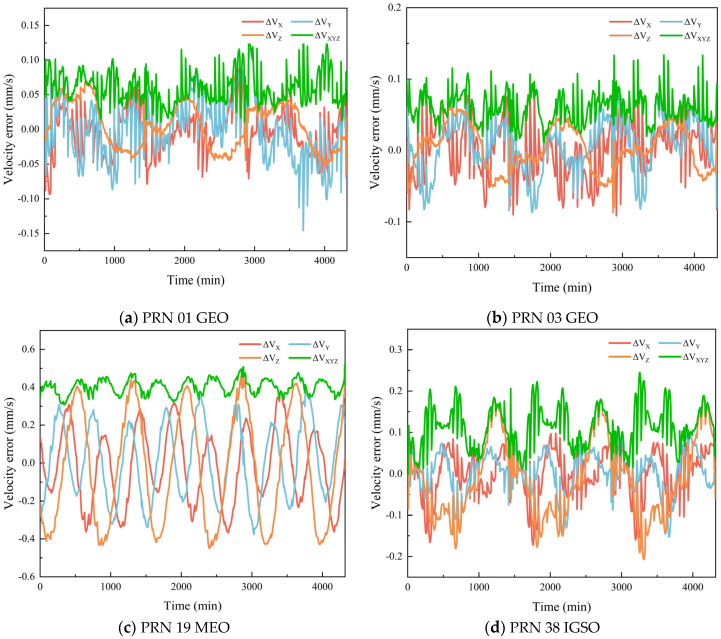
Velocity calculation error of BDS broadcast ephemeris.

Since the BDS-3 constellation consists of three types of satellites, namely MEO, GEO, and IGSO, the position and velocity calculation accuracy is analyzed for all available satellites. Due to the absence of precise ephemeris data for PRN46 (a MEO satellite) in the reference products provided by the Multi-GNSS Data Processing Center of Wuhan University, a total of 29 BDS satellites are finally included in the analysis. To illustrate the temporal evolution of the position and velocity errors, four representative satellites—PRN01 (GEO-1), PRN03 (GEO-3), PRN19 (MEO-1), and PRN38 (IGSO-1)—are selected for detailed presentation. It should be noted that due to the geostationary nature of GEO satellites, they have no explicit ascending node, and an artificial rotation angle is required when generating their ephemeris, resulting in different position and velocity calculation methods compared with MEO and IGSO satellites. Therefore, two GEO satellites are selected to better analyze their position and velocity calculation accuracy. Figure 5 and Figure 6 show the variations of position and velocity errors of four BDS satellites in each direction over a period of one day.

**Figure 7:**
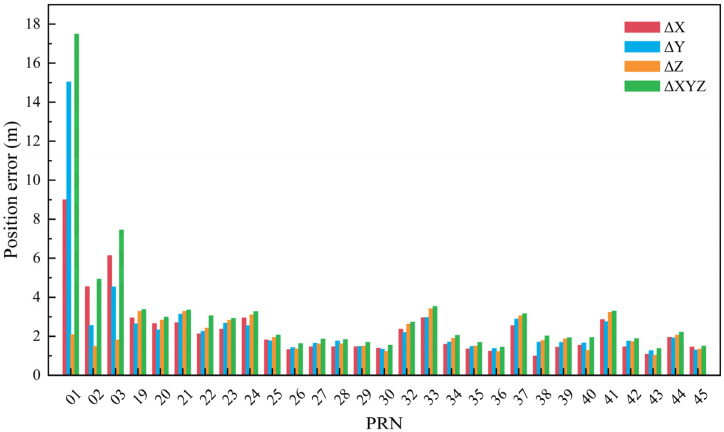
The maximum position errors across the entire constellation over the three-day period.

**Figure 8:**
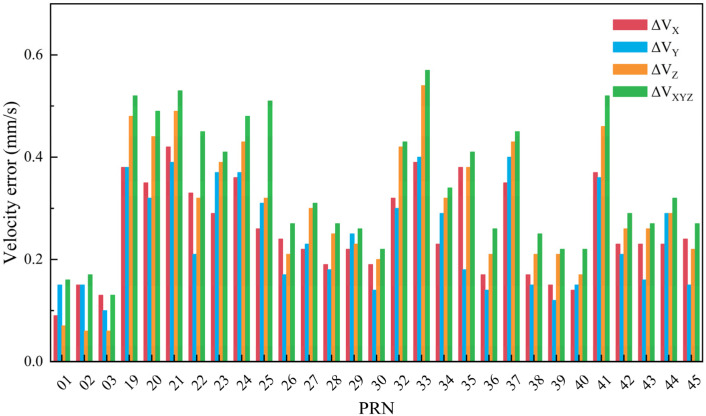
The maximum velocity errors across the entire constellation over the three-day period.

The maximum position and velocity errors across the entire constellation over the three-day period are presented in Figure 7 and Figure 8. In these figures, PRN01, PRN02, and PRN03 denote GEO satellites, PRN38, PRN39, and PRN40 denote IGSO satellites, and the remaining PRNs correspond to MEO satellites.

The position and velocity error values for each satellite shown in Figure 7 and Figure 8 are summarized in Table 1. Based on the analysis of the experimental results, the following conclusions can be drawn:

(1) The maximum position calculation error for BDS MEO and IGSO satellites does not exceed 4 m. In contrast, GEO satellites exhibit larger position errors, with the X and Y directions being the dominant contributors. Among them, GEO-01 satellite has the largest position error, reaching a maximum of approximately 17.5 m.
(2) The velocity error of BDS GEO satellites derived from broadcast ephemeris is the smallest, and can be controlled within 0.2 mm/s. For MEO satellites, the velocity error can be controlled within 0.6 mm/s. For IGSO satellites, the velocity error can be controlled within 0.3 mm/s.

### 4.2. Fast Acquisition Simulation and Analysis

#### 4.2.1. Computational Complexity Comparison of Acquisition Strategies

Based on the analysis of BDS broadcast ephemeris accuracy, the inter-satellite acquisition strategy can be discussed. When both link-establishing satellites are MEO or IGSO satellites, the maximum position errors of MEO and IGSO satellites are approximately 4 m, respectively, and the velocity error is no more than 0.6 mm/s. Taking a threefold redundancy, the coordinate and velocity calculation errors can be set to 12 m and 1.8 mm/s, respectively. Assuming that the errors are fully projected onto the line-of-sight direction, the time delay uncertainty interval is calculated to be approximately 0.4 chips, and the Doppler frequency uncertainty interval is approximately 0.1 Hz. Since the time delay uncertainty is less than half a chip and the Doppler frequency uncertainty is far smaller than the pull-in range of the tracking stage, the signal acquisition phase can be skipped and the receiver can directly enter the tracking phase.

When a GEO satellite is involved in the link establishment, the maximum position error of the GEO satellite is approximately 17.5 m, and the velocity error is no more than 0.6 mm/s. Taking a threefold redundancy, the coordinate and velocity calculation errors can be set to 52.5 m and 1.8 mm/s, respectively. The calculated time delay uncertainty interval is approximately 1.8 chips, and the Doppler frequency uncertainty interval is approximately 0.1 Hz. Since the time delay uncertainty is larger than half a chip, the acquisition phase cannot be skipped in this case, and signal acquisition must be performed before entering the tracking phase. Since Doppler frequency search is unnecessary, we have $N_{f_{d}}=1$. Taking the time delay search step size as half a chip, the number of search bins is calculated to be $N_{\tau }=4$. The computational complexity of the serial and code-parallel acquisition algorithms under this condition are summarized in Table 2.

First, the multiplication complexity of serial acquisition and code-parallel acquisition is compared. Let:

(8) $$f_{multi}(t)=M[(6t\log_{2}t+8t)-4(4t+2)]$$

where t≥1 and M is a positive integer. The derivative of is given by:

(9) $${f^{\prime }}_{multi}(t)=M(3\log_{2}t+\frac{3}{\ln2}-4)$$

For t≥1, ${f^{\prime }}_{multi}(t)>0$, indicating that $f_{multi}(t)$ is monotonically increasing. When t=4, $f_{multi}(4)>0$. Obviously, the number of sampling points for ISL signal acquisition is far greater than 4, so $f_{multi}(t)>0$, meaning that the multiplication complexity of code-parallel acquisition is greater than that of serial acquisition.

Similarly, the addition complexity of the two algorithms can be compared. Let:

(10) $$f_{add}(t)=M[(12t\log_{2}t+4t)-8t]-t+4$$

Following the same analysis, when t≥2, it can be shown that the addition complexity of code-parallel acquisition is also greater than that of serial acquisition. Therefore, based on BDS navigation ephemeris assistance, serial acquisition has the lowest computational complexity and can be selected as the optimal strategy for BDS-3 ISL signal acquisition.

#### 4.2.2. Acquisition Results and Analysis

In this subsection, simulation experiments are designed to evaluate the acquisition performance of the selected inter-satellite link signal acquisition strategy. The simulation parameters are first provided, as detailed in Table 3.

**Figure 9:**
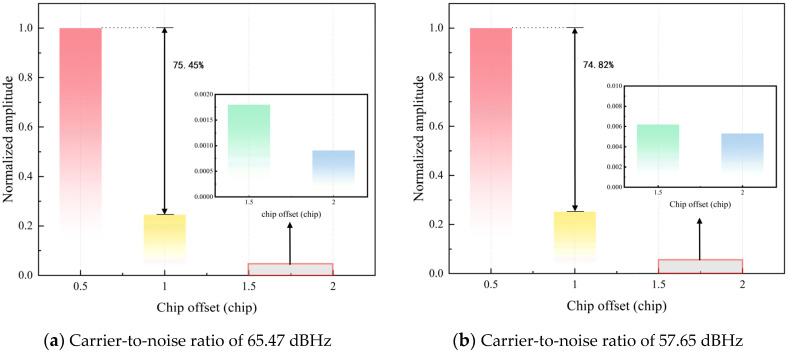
Inter-satellite Acquisition Results Diagram Assisted by BDS Broadcast Ephemeris.

Considering the actual ISL operating scenario, factors such as signal transmission power, antenna gain, free-space propagation loss, pointing loss, feeder loss, and system margin are taken into account. When the inter-satellite distance is at its maximum, the carrier-to-noise ratio is approximately 57.65 dBHz; when the distance is at its minimum, the carrier-to-noise ratio is approximately 65.47 dBHz. Experiments are conducted under these two carrier-to-noise ratio conditions. The number of simulation runs is set to 100, and the results are averaged and normalized. Figure 9 shows the acquisition results. It can be observed that the acquisition results under the two carrier-to-noise ratio conditions are similar. When the time delay error is between 0.5 and 1 chip, the resulting amplitude is approximately 75% lower than that within half a chip. When the time delay error exceeds 1 chip, the amplitude is significantly lower than that within half a chip.

The detailed experimental results are shown in Table 4. Under both carrier-to-noise ratio conditions, the experiment achieves a 100% acquisition success rate, which fully demonstrates that the proposed acquisition method has good acquisition performance in the scenario considered in this experiment. During the entire acquisition process, the coherent integration duration is 0.1 ms, the number of non-coherent accumulations is 5, and the number of code phase search bins is 4. Therefore, the total time consumption is approximately 2 ms, which meets the requirements for fast inter-satellite acquisition.

### 4.3. Ranging Performance Analysis

In this subsection, the ranging performance of the proposed IBFD architecture is evaluated and compared with the traditional TDHD architecture. The objective is to verify that the proposed method achieves a 5-fold improvement in communication efficiency while maintaining the ranging accuracy essentially unchanged.

Table 5 presents the parameter table for inter-satellite ranging performance analysis. Based on the simulation analysis of inter-satellite acquisition performance, the acquisition time assisted by BDS broadcast ephemeris can be reduced to the millisecond level. To reserve a certain engineering margin and account for potential issues such as ephemeris accuracy degradation, the inter-satellite acquisition duration is set to 50 ms. Consequently, the remaining preamble duration available for inter-satellite tracking and measurement is 0.2 s.

In this experiment, the carrier-to-noise ratio is set to the worst-case condition corresponding to the maximum inter-satellite distance. Under this condition, the carrier-to-noise ratio is 57.65 dBHz for the IBFD architecture and 54.65 dBHz for the TDHD architecture. The 3 dB difference arises because the IBFD architecture adopts BPSK modulation for signal tracking, which provides a 3 dB improvement over the QPSK modulation used in the TDHD architecture. Since the signal tracking duration in the IBFD architecture is reduced from 1 s to 0.2 s, a larger code loop equivalent noise bandwidth $B_{n}$ is required. In this paper, $B_{n}$ is set to 12 Hz for the IBFD architecture and 6 Hz for the TDHD architecture, based on the criterion of achieving good simulation convergence performance.

**Figure 10:**
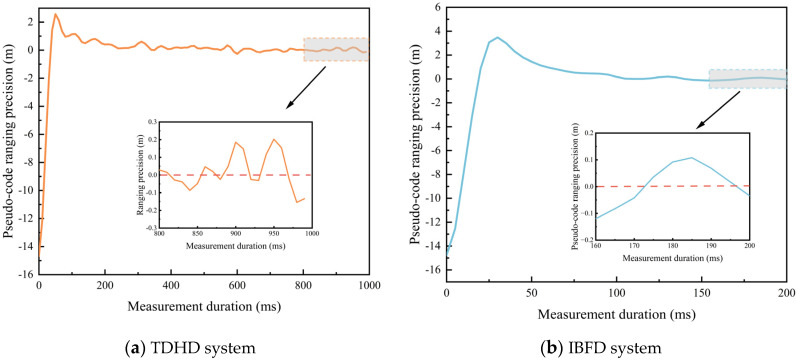
The signal tracking curves of the two architectures.

Figure 10 shows the signal tracking curves of the two architectures. The last 20% of the total duration of each curve is used for measurement accuracy analysis. The simulation results indicate that the ranging accuracy of the IBFD architecture is approximately 9.19 cm, while that of the TDHD architecture is approximately 9.54 cm. The measurement accuracies of the two architectures are essentially consistent.

To further verify the correctness of the experimental results, the theoretical measurement accuracy under the simulation parameters of both architectures is calculated. The formula for calculating the pseudorange measurement error is given as follows:

(11) $$\sigma_{tDLL}=\frac{1}{R_{c}}\sqrt{\frac{2d^{2}B_{n}}{C/N_{0}}[2(1-d)+\frac{4d}{T_{\mathrm{coherent}}C/N_{0}}]}$$

According to Equation (11), the pseudorange measurement accuracy is calculated to be 9.42 cm for both the IBFD and TDHD architectures, which verifies the correctness of the simulation results. Based on the analysis in this section, it can be concluded that improving the carrier-to-noise ratio by modifying the traditional modulation scheme can compensate for the error caused by the accuracy loss in the code loop equivalent noise bandwidth. This demonstrates that the proposed architecture can effectively improve the inter-satellite communication efficiency by up to 5 times while maintaining the inter-satellite measurement accuracy.

## 5. Discussion

Based on the IBFD technology, this paper proposes a measurement and communication cooperative optimization architecture for ISLs. However, the above analysis is mainly conducted under ideal conditions and lacks a discussion of the method’s robustness under non-ideal scenarios, which is addressed as follows.

The proposed BDS ephemeris-assisted fast signal acquisition method uses real-time broadcast ephemeris and does not consider the scenario where ephemeris aging leads to accuracy degradation. Assuming a tenfold degradation in ephemeris accuracy, i.e., a maximum position error of approximately 500 m and a maximum velocity error of approximately 20 mm/s, the time delay uncertainty is calculated to be about 20 chips, and the Doppler frequency uncertainty is on the order of Hz. Based on the preceding analysis, with a search step of 0.5 chips, the total number of time-domain searches is approximately 40, which can be completed within 20 ms. Since the signal acquisition duration is set to 50 ms in this paper, the proposed method remains robust and capable of completing acquisition within the specified time even under ephemeris aging conditions. When a satellite undergoes an orbital maneuver, the ground control segment typically issues a maneuver notification in advance or in real time. During the maneuver, the satellite trajectory deviates from the broadcast ephemeris, and the proposed ephemeris-assisted acquisition method may become ineffective. In this case, the system can switch to the conventional acquisition mode based on ground commands or autonomous detection. Once the maneuver is completed and the satellite resumes normal broadcast ephemeris, the system can switch back to the proposed high-efficiency measurement mode. In summary, the proposed method exhibits strong robustness under ephemeris aging scenarios and provides a feasible fallback mechanism for orbital maneuver scenarios.

**Figure 11:**
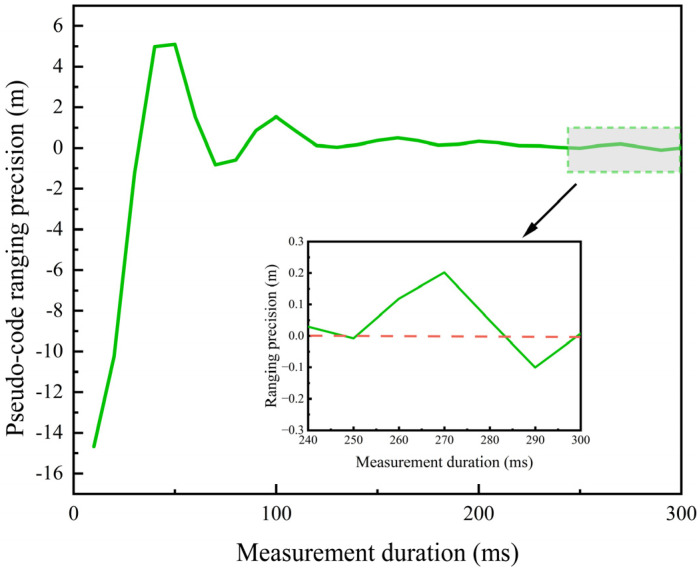
Ranging accuracy under ideal and non-ideal conditions in IBFD.

It should be noted that the ranging performance analysis in this paper is based on ideal conditions and does not account for various non-ideal factors in real on-orbit environments, such as thermal cycles and antenna deformations that may cause SIC performance degradation, residual self-interference inherent in full-duplex operation that was not included as a noise term in the link budget, and local oscillator out-of-phase noise that can introduce additional phase jitter. To better reflect actual on-orbit conditions and address these concerns, this subsection assumes that the combined effect of these non-ideal factors results in an approximately 1 dB degradation in the received carrier-to-noise ratio. Under ideal conditions, when carrier-to-noise ratio is 57.65 dB, the ranging accuracy is 9.18 cm. After considering the 1 dB degradation, carrier-to-noise ratio decreases to 56.65 dB, and the ranging accuracy degrades accordingly. To compensate for this loss, the tracking duration can be extended from 200 ms to 300 ms, while reducing the code loop equivalent noise bandwidth from 12 Hz to 10 Hz. Under this configuration, the ranging accuracy is restored to 9.18 cm. The simulation results are shown in Figure 11. With this setup, the dual-channel communication duration within the 2.5 s time slot is 2.4 s, and the communication efficiency improvement ratio decreases from 5 times under ideal conditions to 4.8 times. Nevertheless, compared with the traditional TDHD scheme, the 4.8-fold improvement remains highly significant, demonstrating that the proposed method maintains robust ranging accuracy and substantial communication efficiency gains even under the influence of multiple non-ideal factors.

It should be particularly noted that the communication efficiency analysis in this paper does not account for protocol overhead. Since the specific frame structure of BDS-3 ISLs is not publicly available, the protocol overhead is not deducted when calculating the communication efficiency improvement ratio. Both the traditional TDHD scheme and the proposed IBFD scheme are compared under the same framework, and the protocol overheads of both schemes are not deducted, ensuring a consistent comparison basis. In practical engineering applications, if the protocol overheads of the two schemes are comparable, the claimed 5-fold improvement in communication efficiency provides a reasonable reference value.

## 6. Conclusions

This paper proposed a cooperative design of ranging and communication for inter-satellite links based on IBFD architecture. The main contributions and findings of this work are summarized as follows.

(1) A fast inter-satellite acquisition method assisted by BDS broadcast ephemeris was developed. The uncertainty regions of time delay and Doppler frequency were determined based on the position and velocity accuracy of MEO, IGSO, and GEO satellites. For MEO and IGSO satellites, the time delay uncertainty is less than 0.5 chips, allowing the acquisition phase to be skipped. For scenarios involving GEO satellites, serial acquisition was selected as the optimal strategy. Simulation results demonstrate that a 100% acquisition success rate is achieved within 2 ms, releasing the 250 ms preamble for other purposes.
(2) A time-slot reconfiguration scheme under the IBFD architecture was designed. Keeping the 3 s time slot unchanged, the proposed architecture divides it into a 0.25 s BPSK acquisition and tracking period, a 2.5 s QPSK dual-channel communication period, and a 0.25 s guard interval. The BPSK tracking provides a 3 dB improvement in carrier-to-noise ratio, compensating for the reduced tracking duration. The QPSK dual-channel communication extends the effective communication duration from 1 s to 2.5 s and expands it from single to dual channel, theoretically achieving a 5-fold improvement in communication efficiency. Both simulation and theoretical results confirm that the ranging accuracy remains essentially unchanged.

Future work will focus on the following two aspects. First, on-orbit validation of the proposed IBFD architecture will be carried out using actual BDS-3 satellites or dedicated testbeds to verify its feasibility and performance in real space environments. Second, based on the IBFD architecture, further research will be conducted on autonomous orbit determination, inter-satellite time synchronization, and routing networking to further enhance the service performance of the BDS-3 system.

## Figures and Tables

**Table 1:** The position and velocity error values for each satellite.

PRN	Position Error (m)				Velocity Error (mm/s)			
	X	Y	Z	3D	X	Y	Z	3D
01	9	15.04	2.08	17.49	0.09	0.15	0.07	0.16
02	4.54	2.55	1.49	4.93	0.15	0.15	0.06	0.17
03	6.13	4.53	1.81	7.45	0.13	0.1	0.06	0.13
19	2.94	2.64	3.27	3.37	0.38	0.38	0.48	0.52
20	2.65	2.33	2.82	2.98	0.35	0.32	0.44	0.49
21	2.7	3.14	3.27	3.34	0.42	0.39	0.49	0.53
22	2.12	2.25	2.41	3.05	0.33	0.21	0.32	0.45
23	2.37	2.67	2.81	2.91	0.29	0.37	0.39	0.41
24	2.94	2.54	3.1	3.26	0.36	0.37	0.43	0.48
25	1.81	1.76	1.94	2.06	0.26	0.31	0.32	0.51
26	1.31	1.42	1.35	1.62	0.24	0.17	0.21	0.27
27	1.46	1.64	1.6	1.86	0.22	0.23	0.3	0.31
28	1.46	1.75	1.6	1.83	0.19	0.18	0.25	0.27
29	1.46	1.48	1.5	1.68	0.22	0.25	0.23	0.26
30	1.38	1.33	1.22	1.55	0.19	0.14	0.2	0.22
32	2.37	2.19	2.62	2.73	0.32	0.3	0.42	0.43
33	2.95	2.96	3.41	3.53	0.39	0.4	0.54	0.57
34	1.59	1.7	1.89	2.04	0.23	0.29	0.32	0.34
35	1.35	1.48	1.5	1.68	0.38	0.18	0.38	0.41
36	1.23	1.37	1.21	1.44	0.17	0.14	0.21	0.26
37	2.54	2.88	3.05	3.16	0.35	0.4	0.43	0.45
38	0.99	1.69	1.78	2.01	0.17	0.15	0.21	0.25
39	1.44	1.68	1.87	1.93	0.15	0.12	0.21	0.22
40	1.55	1.65	1.27	1.94	0.14	0.15	0.17	0.22
41	2.85	2.75	3.22	3.29	0.37	0.36	0.46	0.52
42	1.46	1.75	1.72	1.88	0.23	0.21	0.26	0.29
43	1.08	1.26	1.03	1.37	0.23	0.16	0.26	0.27
44	1.95	1.92	2.05	2.21	0.23	0.29	0.29	0.32
45	1.45	1.29	1.34	1.5	0.24	0.15	0.22	0.27

**Table 2:** Comparison of computational complexity between serial acquisition and code-parallel acquisition algorithms.

Acquisition Algorithm	Multiplication Operations	Addition Operations
Serial acquisition	4(4N+2)M	4(2NM−1)
Code-parallel acquisition	$(6N\log_{2}N+8N)M$	$(12N\log_{2}N+4N)M-N$

**Table 3:** Parameter table for inter-satellite rapid acquisition simulation.

Parameter	Description	Value	Unit
$f_{c}$	Carrier frequency	23	GHz
$R_{c}$	Spreading code rate	10.23	MHz
$L_{c}$	Spreading code length	1023	chip
$T_{\mathrm{coh}}$	Coherent integration duration	0.1	ms
Δτ	Time delay uncertainty range	2	chip
$\Delta f_{d}$	Doppler frequency uncertainty range	0.1	Hz
$\tau_{\mathrm{step}}$	Code phase search step size	0.5	chip
M	Number of non-coherent accumulations	5	\
K	Monte Carlo simulation runs	100	\

**Table 4:** Inter-satellite Acquisition Experiment Results Assisted by BeiDou Broadcast Ephemeris.

Carrier-to-Noise Ratio (dBHz)	Acquisition Results (Normalized Amplitude)				Success Rate (%)
	0–0.5 Chip	0.5–1 Chip	1–1.5 Chips	1.5–2 Chips	
65.47	1	0.2455	0.0018	0.0009	100
57.65	1	0.2518	0.0062	0.0053	100

**Table 5:** Parameter table for inter-satellite ranging performance analysis.

Parameter	Description	Value		Unit
		IBFD System	TDHD System	
$T_{\mathrm{all}}$	The duration of the signal tracking	0.2	1	s
$B_{n}$	Equivalent noise bandwidth of code loop	12	6	Hz
d	Correlator interval	0.5	0.5	\
$T_{\mathrm{coherent}}$	Coherent integration duration	2	2	ms
$B_{L}$	Equivalent noise bandwidth of carrier loop	25	25	Hz
$T_{\mathrm{non}\text{\_}\mathrm{coh}}$	Incoherent cumulative number	5	5	\
$G_{carrier}$	Carrier loop gain	0.01	0.01	\
$G_{code}$	Code loop gain	0.01	0.01	\
$C/N_{0}$	Carrier-to-noise ratio	57.65	54.65	dBHz

## Data Availability

The original contributions presented in this study are included in the article. Further inquiries can be directed to the corresponding author.

## References

[1] Geng T., Han K., Xie X., Wang X., Zhang F. (2023). Estimating Inter-Satellite Link Ka-Band Antenna Phase Center Offsets to Improve BDS-3 Satellite Orbit Determination. Adv. Space Res..

[2] Wang D.X., Guo R., Liu L., Yuan H., Li X.J., Pan J.Y., Tang C.P. (2022). A Method of Whole-Network Adjustment for Clock Offset Based on Satellite-Ground and Inter-Satellite Link Observations. Remote Sens..

[3] Li L.L., Geng G.T., Li Z.H. (2016). Study of the Development of the Inter-Satellite Links in Foreign GNSS. J. Zhengzhou Inst. Surv. Mapp..

[4] Du Y.H., Wang L., Xing L.N., Yan J.G., Cai M.S. (2021). Data-Driven Heuristic Assisted Memetic Algorithm for Efficient Inter-Satellite Link Scheduling in the BeiDou Navigation Satellite System. IEEE/CAA J. Autom. Sin..

[5] Xiao W., Wu Z., Li Z., Fan L., Guo S., Chen Y. (2025). Research on the Autonomous Orbit Determination of Beidou-3 Assisted by Satellite Laser Ranging Technology. Remote Sens..

[6] Cheng J.L., Liu W.K., Zhang X.H., Wang F.H., Li Z.Q., Tang C.P., Pan J.Y., Chang Z.Q. (2023). On-Board Validation of BDS-3 Autonomous Navigation Using Inter-Satellite Link Observations. J. Geod..

[7] Yang Z.M., Yuan Y.B., Tan B.F. (2025). A Short-Arc Hardware Delay Estimation Method for Inter-Satellite Links to Improve BDS-3 Precise Orbit Determination. Sci. Rep..

[8] Li X.B., Yang Z.X., Wang K., Cao L., Qin J.Y., Tang M. (2022). Integrated Signal Dynamic Power Distribution Method for Inter-Satellite Ranging and Communication Links in Navigation Constellation. Adv. Space Res..

[9] Yang Y., Liu L., Li J., Yang Y., Zhang T., Mao Y., Sun B., Ren X. (2021). Featured Services and Performance of BDS-3. Sci. Bull..

[10] Yang Y., Gao W., Guo S., Mao Y., Yang Y. (2019). Introduction to BeiDou-3 Navigation Satellite System. Navigation.

[11] Lv Y., Geng T., Zhao Q.L., Xie X., Zhang F., Wang X. (2020). Evaluation of BDS-3 Orbit Determination Strategies Using Ground-Tracking and Inter-Satellite Link Observation. Remote Sens..

[12] Gong X., Huang D., Cai S., Zhou L., Yuan L., Feng W. (2019). Parameter Decomposition Filter of BDS-3 Combined Orbit Determination Using Inter-Satellite Link Observations. Adv. Space Res..

[13] Zhou W., Cai H., Li Z., Tang C., Hu X., Liu W. (2022). Research on the Rotational Correction of Distributed Autonomous Orbit Determination in the Satellite Navigation Constellation. Remote Sens..

[14] Zhu J., Li H.N., Li J., Ruan R.G., Zhai M. (2022). Performance of Dual One-Way Measurements and Precise Orbit Determination for BDS via Inter-Satellite Link. Open Astron..

[15] Zhou Y., Wang Y., Huang W., Yang J., Sun L. (2018). In-Orbit Performance Assessment of BeiDou Intersatellite Link Ranging. GPS Solut..

[16] Geng T., Yan K., Xie X., Zhang C., Zhang F., Wang X., Chen H.L., Zhao Q.L., Liu J.N. (2025). Two Strategies to Estimate Inter-Satellite Link Hardware Delays in BDS-3 Precise Orbit and Clock Determination: Comparison and Cross-Check. J. Geod..

[17] Zhang C., Geng T., Xie X., Zhao Q., Li T., Li Z., Meng Y. (2024). Analysis of BDS Inter-Satellite Link Ranging Performance. Adv. Space Res..

[18] Guo Y., Zeng L., Zhang F., Bai Y., Chen X., Gao Y., Zou D., Lu X. (2024). Time Synchronization between Satellites via Inter-Satellite Link Observations of BDS-3 Constellation: Method, Experiment and Analysis. Measurement.

[19] Li X., Guo R., Chen G., Zhou S., Sha H., Ma Q., Zhao Y., Zhang L., Wu S., Guo J. (2025). Space–Ground Joint Support Method in Autonomous Orbit Determination of BeiDou Satellites. Remote Sens..

[20] Xia F., Zhou S., Li Z., Jiang N., Hu X. (2024). Analysis of Long-Term Distributed Autonomous Orbit Determination for BeiDou-3 Satellites. J. Geod..

[21] Yan K., Xie X., Geng T., Zhang C., Li Q., Xiong F., Zhao Q. (2026). Autonomous Clock Synchronization and Timekeeping of BDS-3 Constellation Based on Intersatellite Link Measurements. IEEE Trans. Instrum. Meas..

[22] Guo Y., Bai Y., Zhang J., Li W., Zou D., Gao S., Yuan H., Gao Y., Lu X. (2023). Methods and Assessments of Two-Way Time Synchronization Based on BDS-3 Ka-Band Satellite-Ground Link Observations. GPS Solut..

[23] Wang N., Sun L., Fang Y., Lu Z., Ding Q., Wang C., Huang W. (2025). Assignment of Hybrid Laser and Microwave Inter-Satellite Links for Navigation Satellite Systems. Sci. Rep..

[24] Yan J., Song G., Leus R., Hou Z., Zhang Z. (2022). Rolling Weight-Matching Methods for the Inter-Satellite Link Assignment in Global Navigation Satellite Systems. GPS Solut..

[25] Li X.J., Hu X.G., Guo R., Tang C.P., Liu S., Xin J., Guo J.L., Tian Y.J., Yang Y.F., Yang J.H. (2023). Precise Orbit Determination for BDS-3 GEO Satellites Enhanced by Intersatellite Links. GPS Solut..

[26] Kolodziej K.E., Perry B.T., Herd J.S. (2019). In-Band Full-Duplex Technology: Techniques and Systems Survey. IEEE Trans. Microw. Theory Techn..

[27] Feng H., Ma H., Jiao Y., Li X. (2025). Co-time co-frequency full duplex: Techniques and status. J. Space Eng. Univ..

[28] Rao J., Ming Z., Zhang J., Li Z., Chiu C.Y., Murch R. (2025). A Compact Shared-Aperture Antenna with 2-Transmit and 2-Receive Highly-Isolated Ports for Full-Duplex MIMO Systems. IEEE Open J. Antennas Propag..

[29] Zhou J., Shen Y., Xue Y.-J., Li L. (2018). Analysis of RF Feedback Chain Isolation in Wireless Co-Time Co-Frequency Full Duplex. J. Electron. Sci. Technol..

[30] Hua Y., Liang P., Ma Y., Cirik A.C., Gao Q. (2012). A Method for Broadband Full-Duplex MIMO Radio. IEEE Signal Process. Lett..

[31] Feng H., Yang Z., Ma H., Wu T., Jiao Y., Chen Q. (2026). Analysis and Simulation of Topology Characteristics of Beidou Inter-Satellite Link Based on STK. J. Geod. Geodyn..

[32] Feng H., Yang Z., Ma H., Jiao Y., Wu T., Ma H., Chen Q. (2025). A New Measurement Method for BDS Inter-Satellite Link Based on Co-Frequency Co-Time Full Duplex System. Sensors.

[33] Li X. (2015). Research on Key Technologies of Inter-Satellite Precise Ranging for Navigation Constellation. Ph.D. Dissertation.

[34] Zhou F., Zhao L., Li L., Hu Y., Jiang X., Yu J., Liang G. (2022). GNSS Signal Acquisition Algorithm Based on Two-Stage Compression of Code-Frequency Domain. Appl. Sci..

[35] Sun W., Kong Y., Duan S.L., Ding W. (2017). A Fast Capture and Tracking Algorithm for Inter-satellite Link between BeiDou Navigation Satellite System Based on Parallel Processing. Chin. J. Sens. Actuators.

[36] Hu T., Liu T., Zhang X., Meng Y., Zhao W., Xu L., Wang Y. (2025). Review of key technologies in the Inter-Satellite link of the beidou navigation system. Space Electron. Technol..

[37] Chen J., Zhou Y., Yang J. (2021). Principles of Navigation Satellite System Inter-Satellite Link Measurement and Communication.

